# Examining Online Behaviors of Adult-Child and Spousal Caregivers for People Living With Alzheimer Disease or Related Dementias: Comparative Study in an Open Online Community

**DOI:** 10.2196/48193

**Published:** 2023-11-17

**Authors:** Congning Ni, Qingyuan Song, Bradley Malin, Lijun Song, Patricia Commiskey, Lauren Stratton, Zhijun Yin

**Affiliations:** 1 Department of Computer Science Vanderbilt University Nashville, TN United States; 2 Department of Biomedical Informatics Vanderbilt University Medical Center Nashville, TN United States; 3 Center for Genetic Privacy & Identity in Community Settings Vanderbilt University Medical Center Nashville, TN United States; 4 Department of Biostatistics Vanderbilt University Medical Center Nashville, TN United States; 5 Department of Sociology Vanderbilt University Nashville, TN United States; 6 Department of Neurology Vanderbilt University Medical Center Nashville, TN United States; 7 Care and Support Alzheimer’s Association Chicago, IL United States

**Keywords:** Alzheimer disease or related dementias, informal caregivers, adult-child caregivers, spousal caregivers, online community, sentiment analysis, topic modeling, text classification

## Abstract

**Background:**

Alzheimer disease or related dementias (ADRD) are severe neurological disorders that impair the thinking and memory skills of older adults. Most persons living with dementia receive care at home from their family members or other unpaid informal caregivers; this results in significant mental, physical, and financial challenges for these caregivers. To combat these challenges, many informal ADRD caregivers seek social support in online environments. Although research examining online caregiving discussions is growing, few investigations have distinguished caregivers according to their kin relationships with persons living with dementias. Various studies have suggested that caregivers in different relationships experience distinct caregiving challenges and support needs.

**Objective:**

This study aims to examine and compare the online behaviors of adult-child and spousal caregivers, the 2 largest groups of informal ADRD caregivers, in an open online community.

**Methods:**

We collected posts from ALZConnected, an online community managed by the Alzheimer’s Association. To gain insights into online behaviors, we first applied structural topic modeling to identify topics and topic prevalence between adult-child and spousal caregivers. Next, we applied VADER (Valence Aware Dictionary for Sentiment Reasoning) and LIWC (Linguistic Inquiry and Word Count) to evaluate sentiment changes in the online posts over time for both types of caregivers. We further built machine learning models to distinguish the posts of each caregiver type and evaluated them in terms of precision, recall, *F*_1_-score, and area under the precision-recall curve. Finally, we applied the best prediction model to compare the temporal trend of relationship-predicting capacities in posts between the 2 types of caregivers.

**Results:**

Our analysis showed that the number of posts from both types of caregivers followed a long-tailed distribution, indicating that most caregivers in this online community were infrequent users. In comparison with adult-child caregivers, spousal caregivers tended to be more active in the community, publishing more posts and engaging in discussions on a wider range of caregiving topics. Spousal caregivers also exhibited slower growth in positive emotional communication over time. The best machine learning model for predicting *adult-child*, *spousal*, or *other* caregivers achieved an area under the precision-recall curve of 81.3%. The subsequent trend analysis showed that it became more difficult to predict adult-child caregiver posts than spousal caregiver posts over time. This suggests that adult-child and spousal caregivers might gradually shift their discussions from questions that are more directly related to their own experiences and needs to questions that are more general and applicable to other types of caregivers.

**Conclusions:**

Our findings suggest that it is important for researchers and community organizers to consider the heterogeneity of caregiving experiences and subsequent online behaviors among different types of caregivers when tailoring online peer support to meet the specific needs of each caregiver group.

## Introduction

### Background

Dementia is a clinical syndrome that severely impairs a person’s memory, language, and reasoning abilities [1]. Alzheimer disease is the most common cause of dementia, and approximately 6.5 million Americans aged ≥65 years live with Alzheimer disease or related dementias (ADRD) [2]. Owing to a lack of effective treatments, 80% of persons living with dementia are cared for at home by their informal caregivers (eg, family members, friends, or other unpaid caregivers) [3]. This results in tremendous physical, financial, and emotional challenges for these caregivers [4,5], making them known as *invisible second patients* [6]. It has further been shown that informal ADRD caregivers in different kin relationships (eg, adult children, spouses, or children-in-law, which we refer to as *relationships* in this paper for simplicity) differ in their caregiving experience, physical and mental health, and support needs [7,8].

### Existing Literature and Motivation

To date, research on how to support informal ADRD caregivers has focused primarily on the support provided by health professionals [9] (eg, cognitive-behavioral therapy [10], benefit-finding interventions [11], and educational program [12]). Although professional support can improve caregiving skills and emotional well-being, it is challenging to implement it on a large scale because of limitations in workforce availability and financial support [13-15]. At the same time, an increasing body of theoretical and empirical studies indicates that peer-based social support from offline social networks has a positive impact on informal ADRD caregivers [16,17]. For example, the source-need matching theory [18-21] suggests that social support, including informational and emotional support, from caregivers who share similar social backgrounds or caregiving experiences, is more beneficial for stress coping than the support from those who do not. Notably, research has shown that informal ADRD caregivers with shared caregiving experiences can help each other with unique informational and emotional support [22]. However, similar to professional services, offline peer support also faces scalability issues. For example, offline informal ADRD caregivers often report small, high-quality networks of only 4 people on average. By contrast, those with larger networks tend to report experiencing less role strain and better well-being [23].

The integration of the internet into daily life has enabled caregivers to increasingly discuss health-related topics on online platforms [20], including Twitter [24], Facebook [25], and other online communities [26]. In recent research, higher emotional stress and financial hardship of caregivers were found to be the most significant emerging factors associated with increased health-related internet use [27]. Participation in online peer discussions can reduce depressive symptoms [28], improve quality of life [29], and reduce feelings of loneliness [30]. Caregivers seek support and are willing to share experiences and practical information that they believe will assist other caregivers in online environments [31]. In addition, numerous online communities offer the added advantage of facilitating asynchronous discussions and connecting caregivers without the limitations of time and location. These advantages enable ADRD caregivers to receive sufficient social support while fulfilling the time and space obligations of providing care [32]. However, the studies to date that analyzed online caregiving discussions have paid limited attention to the nature of the relationships between informal ADRD caregivers and persons living with dementia. In this respect, it should be recognized that spouses and adult children constitute the 2 largest types of primary informal ADRD caregivers. Spouses regard caregiving as part of their marital obligations, whereas adult children consider such tasks as an important change in their lifestyle [33]. Moreover, spouses tend to be less selective about whether to take on caregiving responsibilities and are at a higher risk of developing health complications compared with adult-child caregivers [34]. By contrast, adult children tend to experience greater family conflict or work disruptions than spouses [33,35]. Given that there are likely differences in caregiving experiences, characterizing the online behaviors of different types of caregivers by relationships can help design customized solutions to online social support that can efficiently assist each type of informal ADRD caregiver.

Most online platforms (eg, Reddit) do not establish specific forums based on each type of caregiver relationship, such that all caregivers discuss topics within the same forum. Even in online communities with separate forums for different types of caregiver relationships (eg, Talking Points managed by the Alzheimer’s Society in the United Kingdom), not every caregiver discloses their relationship with persons living with dementia publicly, and any caregiver can publish their posts in any forum that they believe is appropriate. Considering the massive number of online sources that may contain ADRD caregiving discussions, it is crucial for researchers to develop machine learning algorithms that can efficiently identify the relationship between caregivers and persons living with dementia based on their online posts, which will be a valuable social determinant that can be applied to online social support-based research. Various machine learning algorithms have been developed to extract relevant information from massive, noisy online data to facilitate biomedical research [36], including, but not limited to, predicting mental health status [37], disseminating study information during the COVID-19 pandemic [38], and learning the needs of patients with ovarian cancer and their caregivers [39]. However, most studies in the ADRD caregiving field have either identified online ADRD caregiving discussions [26] or summarized caregiving challenges [35]. Few studies have differentiated caregivers’ relationships with persons living with dementia.

### Objectives

In this study, we aimed to conduct a comprehensive comparison of the online behaviors of the 2 distinct groups of informal ADRD caregivers: adult-child and spousal caregivers. To achieve this goal, we conducted topic analysis, sentiment analysis, and caregiver relationship classification using the large-scale retrospective data collected from ALZConnected [40], an open online community powered by the Alzheimer’s Association for any person affected by ADRD in North America. Our study not only enriches our understanding of the online behaviors of informal ADRD caregivers in online communities but also highlights the potential for improving online support systems, thereby contributing to the advancement of targeted support for informal ADRD caregivers.

## Methods

### Ethical Considerations

This study was exempted from human participants research by the Institutional Review Board of Vanderbilt University Medical Center (221732). The online posts are publicly accessible through the ALZConnected online community. In this paper, we only present results on an aggregated level, and any quotes presented are carefully rephrased to maintain the privacy of the corresponding users [41].

### Data Collection

ALZConnected is a free online community for caregivers and persons living with dementia to post questions, offer and receive support, and make public and private groups around dementia-related topics and issues. ALZConnected allows individuals to read online discussions without registering on the platform. However, to use all the functions in ALZConnected, including publishing a post, individuals must register to become a member on the platform. During registration, a user is asked to provide information about their relationship with persons living with dementia. Registered users may choose to make such information visible to other logged-in online users. ALZConnected has 10 forums, each organizing online discussions into a collection of topic threads. Each thread is initialized with the first post as the *top post* and can contain many other posts called *comments*. We collected data from ALZConnected using a web crawler that we developed based on the *BeautifulSoup* v4.11 python package. The data contain online user interactions that transpired between October 2011 and August 2022. We maintained all users who self-reported as ADRD caregivers and excluded administrators and moderators.

For this study, we extracted all the posts published in three main caregiver forums: (1) Caregivers Forum, (2) Spouse or Partner Caregiver Forum (referred to as Spouses Forum for simplicity), and (3) Caregivers Who Have Lost Someone Forum (referred to as Lost Forum). Although it is unlikely that a caregiver is active online every day, in this study, we define the *active days* of a user as the number of days between the dates when the user published the first and the most recent posts to improve the readability of the paper. In our analysis, we focused primarily on *adult-child* and *spousal caregivers*; all other relationships were categorized as *other caregivers.*

### Activity Statistics

We characterized caregivers’ online activities along several dimensions: (1) posts and relationships, (2) views and comments, and (3) posts and active days. To do so, we first identified all the self-reported relationships and the corresponding post volumes to provide a big picture with respect to all the online caregivers. Second, ALZConnected displays the number of views and comments for each topic thread publicly, which enables us to compare these 2 statistics on a log scale separately for adult-child and spousal caregivers. Reading and posting are the 2 types of essential activities in online communities that can benefit caregivers in different ways. Third, we compared the distributions of the online posts and active days on a log scale separately for adult children and spouses to gain insights into online activities for both short-term and long-term online caregivers. To avoid the logarithm of 0, we incorporated a weak uniform prior in the form of a small pseudo count. Specifically, this was accomplished by adding 1 to each count.

### Structural Topic Modeling

We applied structural topic modeling (STM) v1.3.6 [42], an R package developed by researchers and contributors, to *top posts* to investigate the topic prevalence for adult children and spousal caregivers. In general, a caregiver initiates a topic thread by asking a question or sharing their experience in the top post, whereas other caregivers make comments in the response below. Therefore, focusing on the top posts instead of comments enables an examination of the challenges communicated by these caregivers. Furthermore, focusing on top posts can reduce the bias induced by *super-active users* who published substantially more posts than others. STM allows for the integration of post-level metadata (eg, authorship) into the topic modeling process. In contrast to standard topic modeling methods such as latent Dirichlet allocation, STM can provide better intuition into the dynamics of social representations through a comparative view [43-45].

In our model training, we incorporated caregiver relationships as a binary meta-variable. This variable indicates whether a top post was composed by an adult child or a spouse. More specifically, we extracted the top posts from all the topic threads, removed stop words and special symbols, and dropped the words that appeared <10 times in the data set. To determine the optimal number of topics in STM, we relied on the measures of *exclusivity*, which refers to the distinctiveness of the words with the highest frequencies in the topic, and *semantic coherence* [46], which quantifies how the words in a topic frequently co-occur together in general contexts [42]. We chose the optimal topic number from a predefined list of [5, 10, 15, 20, 25, 30] by finding the one that made a trade-off between the 2 metrics.

To support the interpretation of the modeling results, we summarized each topic with a name and grouped similar topics together. This summary was developed by the authors through a manual review and discussion of the *top words* ranked by their probabilities in each topic and the 5 *posts* with the highest probabilities of each topic. We also compared adult children and spousal caregivers with respect to topic prevalence. The *effect* for each topic was estimated by regressing the proportion of topics on a binary meta-variable that indicates whether the top post was composed by a spousal caregiver as opposed to an adult-child caregiver. Positive (or negative) effects indicated that the corresponding topics were more likely to be posted by spousal (or adult-child) caregivers.

### Sentiment Analysis

Sentiment analysis refers to a common natural language processing technique that applies computational methods to determine whether a given post conveys positive, negative, or neutral emotions or tones. In this study, we define the *relative days* of a post as the number of days since the first post of its author was published. By realigning all the posts along their relative days rather than published dates (eg, October 19, 2022), we analyzed how the sentiment of a caregiver’s posts changes on average as the caregiver interacts with others in the community over time.

In recognition of the measurement bias that can be introduced by applying off-the-shelf models to this data set, we relied upon 2 existing popular sentiment tools to perform the analysis, which we believed could provide better intuition than the application of a single model. The first tool is the Valence Aware Dictionary for Sentiment Reasoning (VADER) version 3.3.2 [47], which is a rule-based model to evaluate the sentiment of a given text [48]. VADER generates a normalized, weighted compound score that ranges between −1, the most extreme negative sentiment, and +1, the most positive sentiment. The second tool is Linguistic Inquiry and Word Count (LIWC) 2015 [49], which calculates the percentage of each linguistic category by mapping the words in a given text into a predefined word list of the linguistic category [50]. This tool has been widely adopted in online content-based research [51]. In this study, we focused on the *negative emotion* category in LIWC.

### Classification

#### Classification Task

After performing the topic and sentiment analysis, we built machine learning models to classify caregiver relationships, which can further disclose the difference in temporal posting behaviors between adult-child and spousal caregivers. To do so, we conducted a *post-level prediction* by predicting the relationship for each post. We then performed a *user-level prediction* by applying a majority voting model to the prediction labels of all the posts of a caregiver. We represented the post-level prediction as a 3-class classification, where each post was an instance and the label was the corresponding author’s self-reported relationship. The latter was mapped into one of the three relationship categories: (1) *adult child*, (2) *spouse*, and (3) *other* caregiver. This multiclass classification design enabled the trained models to be readily applied to any other ADRD forums.

#### Experiment Design

As the post volume of each online caregiver followed a long-tailed distribution (refer to the Results section), including all the available posts in model training would have biased the fitted model to superactive users. Therefore, to balance between including more training data and mitigating the bias induced by the long-tailed distribution, we proposed a training and evaluation procedure as described in the following subsections.

#### Data Preparation

We removed posts with ≤10 words. This decision was based on a manual review of 100 randomly selected posts, which showed that 83% (83/100) were about greeting or gratitude, website URLs, or incomplete sentences. We applied a stratified shuffle split on user level and selected all the posts for 80% (14,729/18,412) of the users as the training data, denoted by *D_train_*, and the remaining posts as test data, denoted by *D_test_*. In doing so, we ensured that the ratio of the user-level labels remained the same in *D_train_* and *D_test_* and there was no overlap in the authorship across the 2 data sets.

#### Models

We created 4 training subsets by selecting no more than *i* posts from each caregiver in *D_train_*, where *i* in [5, 10, 15, 20] as selecting an exact *i* number of posts might result in subsets that were either too small owing to data sparsity or not diverse enough to capture the full range of variability of the data. We used each subset to train a model *M_i_*. We used the Google Pretrained Bidirectional Encoder Representations from Transformers (BERT) fine-tuning model (“bert-base-uncased” in Transformer package v2.8.0) provided by the Hugging Face Transformers as the algorithm. For a fair comparison, we configured each model with an embedding size of 256, batch size of 32, learning rate of 2×10^−5^, and number of training epochs of 10. It should be noted that in addition to the fine-tuned BERT model, we experimented with other models including logistic regression, random forests, and a bidirectional long short-term memory with attention. However, these models did not outperform the BERT model and hence are not presented in this paper.

#### Evaluation

We grouped the caregivers in *D_test_* into 5 bins based on their post volumes: [1, 1], (1, 5], (5, 10], (10, 100], and (100, ∞). For instance, the user bin (1, 1) comprises caregivers who have composed merely 1 post, whereas the user bin (100, ∞) contains those who have contributed >100 posts. We calculated and compared the model performance in each user bin. Specifically, in each user bin, we randomly sampled 80% (2946/3683) of caregivers and used all their posts as a test subset to calculate the macro area under the precision-recall curve (AUPRC), which is preferred when the labels are unbalanced. We repeated the process 10 times and reported the mean and SD of AUPRC. We also reported precision, recall, and F_1_-score for each type of relationship prediction and the overall macro AUPRC by counting the total true positives, false negatives, and false positives.

#### Temporal Trend

To characterize the temporal patterns of the predictability of online posts in determining relationships, we applied the model with the best performance to calculate the predicted probability of the true label for each post. For example, if a post was composed by a spousal caregiver, we estimated the probability that the model predicts the author as a spousal caregiver. In this task, we focused solely on adult children and spousal caregivers and analyzed the predicted probabilities along with the *relative days* for all the caregivers in the test data set.

## Results

### Activity Statistics

#### Overview

Table 1 summarizes the posts, users, and their active days in the 3 ALZConnected forums. In total, we collected 534,205 posts that were published by 18,622 users in the community. Among the 534,205 posts, 56,737 (10.62%) were top posts. Most of the posts were composed in the Caregivers Forum (287,556/534,205, 53.82%) and Spouses Forum (238,068/534,205, 44.56%), whereas only a small proportion (8581/534,205, 1.6%) was posted in the Lost Forum. The users in the Spouses Forum were more active in posting (averaging 50 posts/user) compared with the users in the Caregivers Forum (18 posts/user) and the Lost Forum (12 posts/user), and they also had the largest mean active days of 829 (SD 853) days.

**Table 1:** The number of posts, users, and their active days in the 3 ALZConnected forums.

	Overall	Forum		
		Caregivers	Spouses	Lost
Posts, n (%)	534,205 (100)	287,556 (53.82)	238,068 (44.56)	8581 (1.61)
Top posts, n (%)	56,737 (100)	34,840 (61.41)	20,767 (36.6)	1130 (1.99)
Users, n (%)	18,622 (100)	15,666 (84.13)	4726 (25.38)	692 (3.72)
Posts per user, mean (SD)	29 (275)	18 (158)	50 (355)	12 (37)
Active days, mean (SD)	689 (791)	572 (718)	829 (853)	751 (708)

#### Posts and Relationships

**Figure 1:**
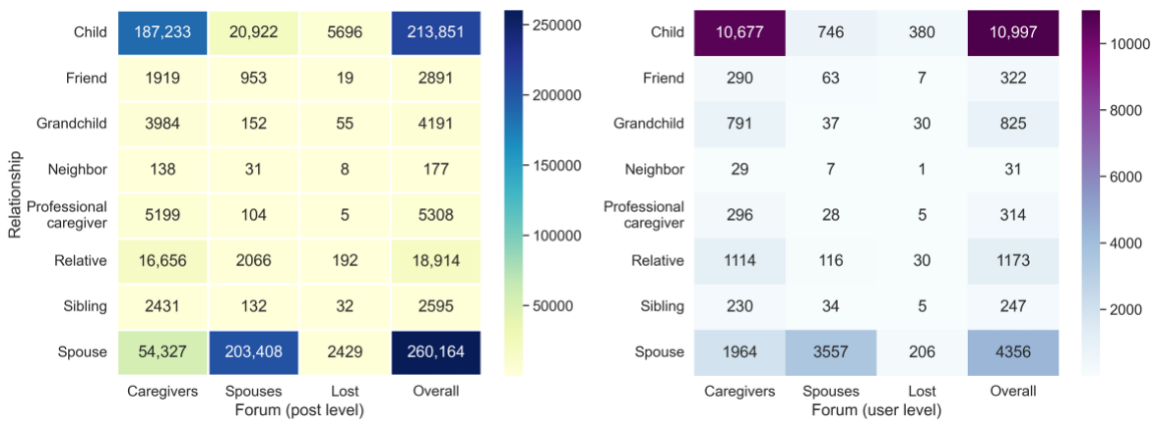
The number of posts (left) and the number of users (right) by relationship types in the 3 forums. The overall columns report the total number of posts (left) and unique users (right).

Figure 1 presents the number of posts (left) and the number of unique caregivers (right) for each type of *self-reported relationship* in the 3 forums. The overall column counts the total number of posts and caregivers in each forum. Adult-child caregivers (10,997/18,265, 60.21%) and spousal caregivers (4356/18,265, 23.85%) constituted most of the users (15,353/18,265, 84.05%) across the 3 forums. Together, they contributed most of the posts (474,015/508,091, 93.29%) in these 3 forums, with 42.1% (213,851/508,091) of adult children posts and 51.2% (260,164/508,091) of spousal posts. This observation was consistent with a previous study that showed that adult children and spouses comprise the 2 two largest proportions of the informal caregivers for persons living with dementia [41].

Although most spousal (or adult children) posts were typically composed in the Spouses (or Caregivers) Forum, we found that a large number of posts were made elsewhere. For example, 45.09% (1964/4356) of the spousal caregivers published 19.98% (54,327/271,887) of the posts in the Caregivers Forum, whereas 6.78% (746/10,997) of the adult-child caregivers generated 9.19% (20,922/227,768) of the posts in the Spouses Forum. In addition, 88.3% (586/664) of the caregivers in the Lost Forum were either adult children or spouses. They composed 96.37% (8125/8431) of the posts in the forum. Other commonly reported relationships were relatives (1173/18,265, 6.42%), grandchildren (825/18,265, 4.51%), friends (322/18,265, 1.76%), siblings (247/18,265, 1.35%), and neighbors (31/18,265, 0.17%), and together they contributed 6.71% (34,076/508,091) of posts in the 3 forums. It should be noted that 1.04% (5308/508,091) of the posts were composed by professional caregivers, which suggested a skewed but diverse ADRD caregiver population in this online community.

#### Views and Comments

**Figure 2:**
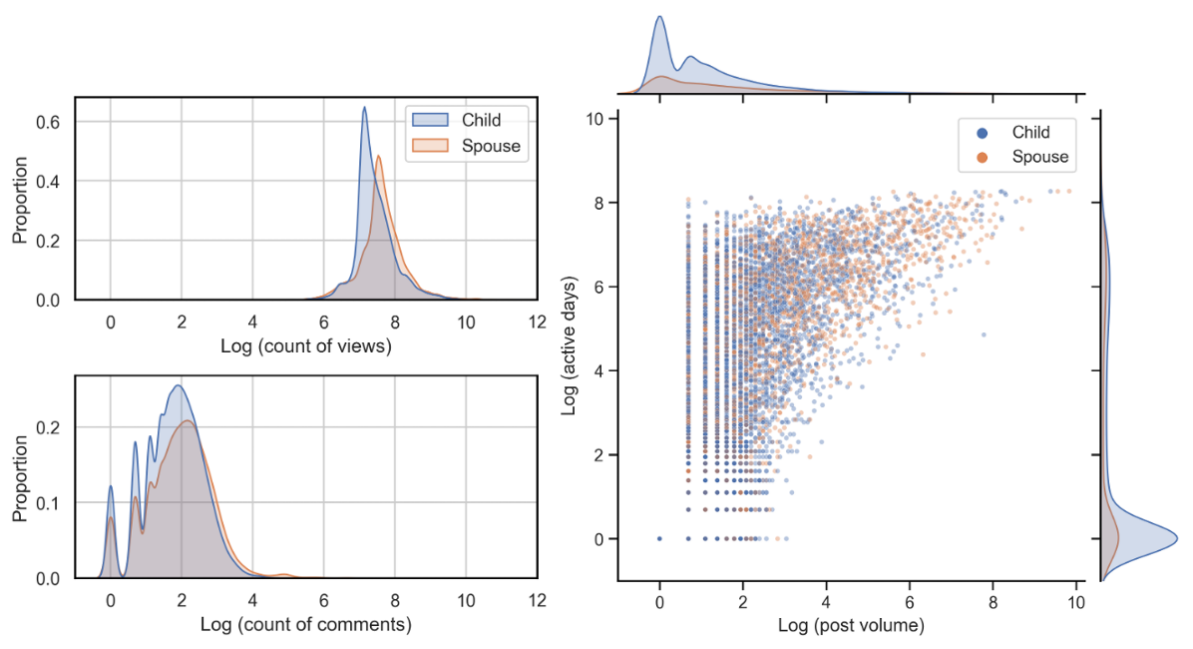
The distribution of comments and views for each topic thread. The x-axis is log transformed for visualization purposes (left); the scatterplot shows posting volume and active days among the caregivers (right). The density plots of both statistics are illustrated along the top and to the right.

Figure 2 (left) compares the views and comments for the topic threads (on a log scale). It was evident that the overwhelming majority of the density for the view of topic threads centered around e^7^≈1100 (with a range of 43-285,450), with spousal topic threads receiving more views than adult children topic threads (under a 2-sample Kolmogorov-Smirnov test, *d*=0.24; *P*<.001). By contrast, the distributions of the comments for both types of caregivers had more modes, and most of the comment numbers were concentrated on 0, 1, and 7 (with a range of 0-2359), which was far less than the views. The 100× difference indicated that the collective knowledge generated in this open community may be consumed by a much larger number of caregivers who either do not have an account or seldom engage in online discussions. A 2-sample Kolmogorov-Smirnov test showed that spousal topic threads received slightly more comments than the adult children topic threads (*d*=0.10; *P*<.001).

#### Posts and Active Days

Figure 2 (right) depicts the scatterplot of post volume and active days for each user (on a log scale). Our study yielded several noteworthy findings. First, both post volume and active days followed a long-tailed distribution, which revealed that only a small number of caregivers published a large number of posts and were active for a long time. For example, the top 1% (109/10,997) of the adult children (spousal, 43/4356, 1%) caregivers with the largest post volume generated 56.52% (120,863/213,851; 124,474/260,164, 47.84%) of all of the adult-child (spousal) posts. The number of posts ranged from 1 to 18,662, whereas the number of active days ranged from 1 to 3899. Second, except for online caregivers who posted only once, most caregivers were short-term online users who published a limited number of posts during a short period. For instance, 89.58% (8519/9510) of the users published <50 posts, and 87.09% (8283/9510) of the users were active for <2 years. Although not shown in Figure 2 (right), 54.82% (4307/7856) of these short-term users (published <50 posts and active for <2 years) were more likely to participate in online discussions within their own topic threads than respond to other caregivers’ topic threads. For these online users, their primary focus might be on addressing specific caregiving challenges or questions. Third, among long-term users who published >50 posts within an >2-year active period, 89.4% (504/564) posted more on other caregivers’ topic threads than on their own topic threads. These caregivers, who are described as “veteran users” of the online community [52], preferred to interact with other online caregivers intensively. Under the 2-sample Kolmogorov-Smirnov test, spousal caregivers tended to publish more posts (*d*=0.11; *P*<.001) and had longer active days (*d*=0.14; *P*<.001).

### Caregiving Topics

The optimal number of topics was determined to be 25. Table 2 presents these topics, along with their most representative words (top words) based on the highest word probabilities in a topic and the expected topic proportion in the entire data that were used to train the STM.

**Table 2:** Summary of the 25 topics. The words in each topic are ranked in decreasing order based on their estimated probabilities in the topic.

Group and topic			Top words		Expected topic proportion
**Daily caring issues**					
	#12: Balanced living	take, home, day, work, will, time, hous, need, come, get		0.056	
	#13: Sleeping	night, day, sleep, bed, idn’t, walk, get, last, pain, hour		0.039	
	#18: Showering	use, clean, get, bathroom, shower, put, cloth, room, floor, wear		0.032	
	#11: Financial issues	pay, money, phone, bill, check, get, insur, idn’t, call, can		0.028	
	#1: Transportation	car, door, walk, open, hous, store, idn’t, drive, light, key		0.028	
	#14: Traveling	dog, get, morn, hope, one, cat, ill, good, today, got		0.022	
	#9: Diets	eat, food, drink, dinner, meal, feed, cook, water, weight, mouth		0.021	
**Disease related**					
	#20: Loss of expression	say, tri, thing, get, tell, talk, ask, someth, time, always		0.053	
	#16: Disease diagnosis	year, ago, month, now, stage, last, sinc, diagnos, past, week		0.053	
	#15: Loss of memory	memori, seem, chang, can, becom, time, often, word, still, even		0.043	
	#24: Nursing home	care, home, facil, nurs, place, move, visit, assist, memori, staff		0.042	
	#21: Medication	doctor, med, take, medic, hospit, day, agit, also, help, week		0.040	
	#10: Last wishes	need, will, care, medic, make, health, issu, doctor, decis, state		0.035	
	#25: Dementia	test, dementia, alzheim, diseas, brain, diagnosi, symptom, neurologist, cognit, result		0.030	
	#17: COVID19	idn’t, death, patient, program, covid, case, famili, provid, may, state		0.026	
**Emotion**					
	#7: Feelings	feel, husband, life, never, diseas, like, hard, ever, know, cri		0.044	
	#2: Gratitude	love, day, time, friend, wife, happi, miss, watch, one, daughter		0.043	
	#3: Blessings	god, will, love, lord, peac, bless, pray, give, can, jesus		0.038	
**Relationship**					
	#8: Relatives	mother, live, father, famili, sister, caregiv, parent, help, dementia, care		0.028	
	#6: Relatives	mom, dad, shes, brother, sister, visit, also, move, want, help		0.020	
**Others**					
	#4: Common verbs	just, know, dont, want, think, cant, realli, like, can, doesn’t		0.082	
	#5: Common questions	thank, help, post, read, caregiv, can, anyon, find, mani, support		0.065	
	#19: Common words	one, good, well, look, like, thing, lot, thought, way, new		0.050	
	#22: Common verbs	said, call, told, got, idn’t, ask, went, today, back, took		0.050	
	#23: Timing	start, back, get, happen, stop, around, turn, head, just, hand		0.032	

Caregiving discussions were mainly focused on 4 primary topic groups: daily caregiving issues, disease related, sentiment and relationship, together with some common verbs in online communications, which we categorized as others. In particular, the disease related topic group had the largest proportion, contributing 32.3% of the discussion. The second largest topic group was about daily caregiving matters, which included #13 (sleeping issues; 3.9%; eg, “My mom didn’t sleep well last night, and now she’s napping during the day. Should I let her nap?”) and #11 (financial issues; 2.8%; eg, “I am now my mom’s social security payee and I had to use my [bank name] to intervene in my mom’s account because she gave the money to a scammer”) and other issues that were commonly reported in the literature [53,54]. The emotion topic group included #7 feelings (4.4%), #2 gratitude (4.3%), and #3 blessings (3.8%), which mainly describe the mixed emotions they experienced. The relationship topic group was embodied by topics #6 (2%) and #8 (2.8%), both of which were about relatives. On the basis of the top words and their related posts, topic #6 was mentioned more in family conflicts, whereas topic #8 was mentioned more in other scenarios.

**Figure 3:**
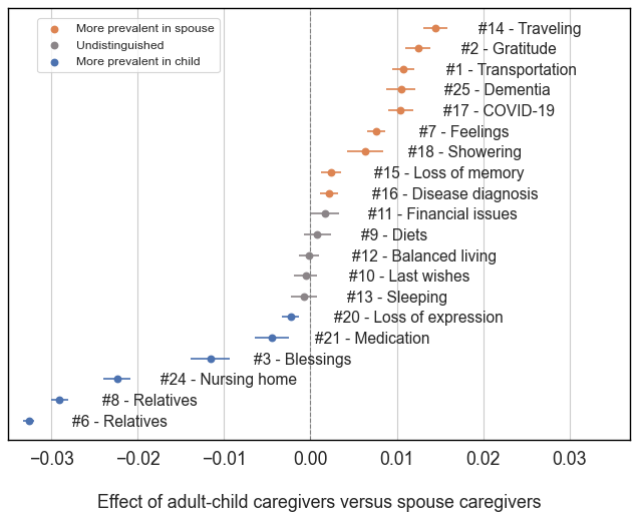
A contrast can be seen in topic prevalence between adult-child and spousal caregivers (with 95% CIs). A positive (negative) value along the x-axis indicates that the topic is more prevalent among spousal (adult-child) caregivers.

Figure 3 compares adult children and spousal caregivers with respect to topic prevalence. There are several findings to highlight. First, although there was a smaller number of spousal caregivers than adult-child caregivers in ALZConnected, spouses discussed a wider range of topics than adult children, including #14 (traveling), #1 (transportation), #25 (dementia), #17 (COVID-19), #18 (showering), #15 (loss of memory), and #16 (disease diagnosis). This aligned well with the literature, which indicated that spousal caregivers report a need to redefine their role and relationship with persons living with dementia [55].

Second, the 2 topics related to #6 and #8 (relatives) and #24 (nursing home) were more frequently discussed by adult children (eg, “my mother will be moving to my sister’s house and she will become my mother’s full time caregiver.... My sister refuses to learn about dementia caregiving. I suggested...move to her house with my mother for a few weeks...she thinks this is unnecessary.”). This suggested that adult children faced more family conflicts when caring for persons living with dementia than spouses [33]. In addition, adult children seemed to discuss the medical topic (#21) more frequently than spouses.

Third, in the emotion topic group, adult children posted content about “praying” in the forums, such as topic #3 blessings (eg, “And I will ask the Father...give you another Helper...to be with you forever”), whereas spouses expressed more personal feeling during daily care, either gratitude or sadness, such as topic #2 gratitude (eg, “I remember when: We laughed together We cried together.... We loved life together We got through tough times together”) and #7 feelings (eg, “I feel guilty...for not being patient with my DH...guilty about mentioning how hard it is to people who really don’t understand and think I’m exaggerating”).

### Sentiment Analysis

**Figure 4:**
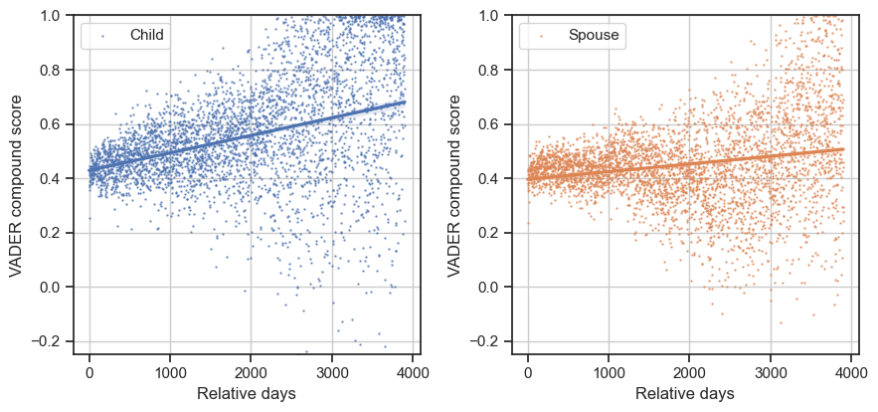
Sentiment in online posts according to the Valence Aware Dictionary for Sentiment Reasoning (VADER) compound sentiment score for adult-child (left) and spousal (right) caregivers. To improve readability, all of 1 day’s scores are averaged into a single point.

**Figure 5:**
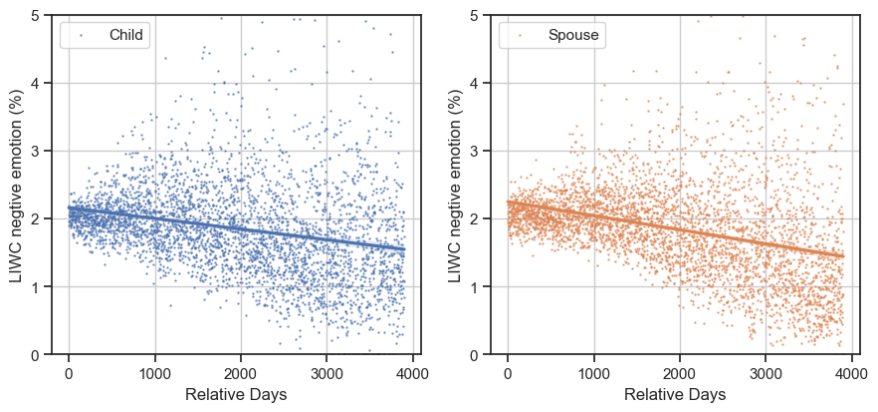
Sentiment in online posts according to the Linguistic Inquiry and Word Count (LIWC) negative emotion score for adult-child (left) and spousal (right) caregivers. To improve readability, all of 1 day’s scores are averaged into a single point.

Figures 4 and 5 illustrate how sentiment changed over time by the VADER compound score and LIWC negative emotion, respectively. To improve readability, we averaged the VADER compound scores or LIWC negative emotion for each of the caregivers during an active day into a single point. The linear interpolation and its 95% CI are shown in Figures 4 and 5.

As shown in Figure 4, which corresponds to the VADER compound score, the average sentiments for both caregiver types were consistently >0.4 and maintained an increasing trend. Compared with spouses, adult children exhibited a higher growth rate in the VADER compound score. The Spearman rank-order correlation between the VADER compound score and *relative days* for adult children and spouses were 0.42 and 0.17 (*P*<.001), respectively. This observation is in agreement with a previous finding that spousal caregivers expressed both gratitude and negative feelings relatively more than adult-child caregivers [56]. Meanwhile, according to the LIWC negative emotion, as shown in Figure 5, both caregiver types exhibited a decrease in negative emotion over time. The Spearman rank-order correlation between the LIWC negative emotion and *relative days* for adult children and spousal caregivers were −0.36 and −0.44 (*P*<.001), respectively. The improved sentiment over time echoes the finding of a prior study that showed that a caregiver’s engagement in an online community is related to a reduction in depressive symptoms [21].

### Classification

#### Overview

After filtering out the posts with ≤10 words, there were 508,030 posts composed by 18,412 caregivers. The ratio of user-level labels was 59:24:17 in class of *adult child* and *spouse* and *other*. The 4 training subsets in *D_train_*, where *i∈*[1,5,10,100], were corresponding to 3.57% (14,729/413,101), 9.42% (38,931/413,101), 13.05% (53,924/413,101), and 33.98% (140,379/413,101) percentile of all the caregivers in *D_train_* based on their post volume. The 5 bins of the test subsets were corresponding to 40.62% (1496/3683; 1496/94,929, 1.58%), 34.21% (1260/3683; 2520/94,929, 2.66%), 12.76% (470/3683; 3675/94,929, 3.87%), 9.04% (333/3683; 14,442/94,929 15.21%), 3.37% (124/3683; 72,796/94,929, 76.68%) of the caregivers (posts) in *D_test_*.

#### Model Performance

Table 3 presents the overall model performance, where *M_i_* represents each model training set, where *i* denotes the maximum number of posts randomly sampled by each user. We want to emphasize several important findings. First, *M_100_* achieved the best AUPRC in post-level prediction 0.737, whereas *M_10_* achieved the best AUPRC in user-level prediction 0.813. Both of them significantly outperformed the second-best model under a 2-tailed *t* test (*P*<.001). Second, owing to the imbalanced labels, all the models had lower model performance in classifying *other* than classifying *adult child* and *spouse*. Third, the models trained using a smaller number of user posts (eg, M_1_ and M_5_) had better performance in predicting *adult child* in both tasks, whereas the models trained using a larger number of user posts (eg, M_10_ and M_100_) had better performance in predicting *Spouse* in both tasks. These observations confirm the necessity of examining model performance in different user bins based on the number of their published posts.

**Table 3:** Performance of prediction at the post level and user level for the machine learning models. All numbers correspond to percentage values.

Task		Post-level prediction (%), mean (SD)					User-level prediction (%),mean (SD)			
		M_1_	M_5_	M_10_	M_100_	M_1_		M_5_	M_10_	M_100_
**Adult child**										
	Precision	61.2 (0.04)	*63.2 (0.04)* ^a^	61.6 (0.04)	62.3 (0.03)	81.4 (0.13)		*86.4 (0.17)*	85.7 (0.19)	85.4 (0.19)
	Recall	*81.8 (0.05)*	76.7 (0.06)	78.7 (0.06)	78.5 (0.04)	*94.3 (0.15)*		92.7 (0.17)	93.4 (0.14)	93.0 (0.18)
	*F*_1_-score	*70.0 (0.04)*	69.3 (0.04)	69.1 (0.04)	69.5 (0.03)	87.4 (0.09)		*89.5 (0.12)*	89.4 (0.12)	89.1 (0.12)
**Spouse**										
	Precision	*83.7 (0.06)*	81.2 (0.03)	81.6 (0.05)	80.9 (0.05)	81.9 (0.40)		81.1 (0.33)	*82.4 (0.38)*	80.2 (0.52)
	Recall	57.7 (0.06)	65.4 (0.60)	64.9 (0.06)	*67.8 (0.07)*	87.5 (0.41)		90.6 (0.34)	*91.4 (0.34)*	91.3 (0.33)
	*F*_1_-score	68.3 (0.05)	72.5 (0.04)	72.3 (0.05)	*73.8 (0.06)*	85.6 (0.26)		85.6 (0.27)	*86.7 (0.25)*	85.4 (0.34)
**Other**										
	Precision	14.8 (0.08)	20.0 (0.10)	21.6 (0.15)	*23.4 (0.12)*	66.6 (0.76)		74.9 (0.54)	*77.6 (0.58)*	76.3 (0.46)
	Recall	20.9 (0.14)	*25.0 (0.16)*	21.7 (0.15)	18.8 (0.12)	24.8 (0.49)		*44.6 (0.78)*	42.6 (0.79)	39.5 (0.68)
	*F*_1_-score	17.3 (0.10)	*22.2 (0.13)*	21.7 (0.15)	20.9 (0.12)	36.1 (0.60)		*55.9 (0.70)*	55.0 (0.75)	52.0 (0.66)
AUPRC		71.6 (0.04)	72.8 (0.04)	72.8 (0.05)	*73.7 (0.05)*	75.9 (0.18)		80.6 (0.25)	*81.3 (0.21)*	80.4 (0.26)

^a^The best performance for each metric is highlighted is italicized.

**Figure 6:**
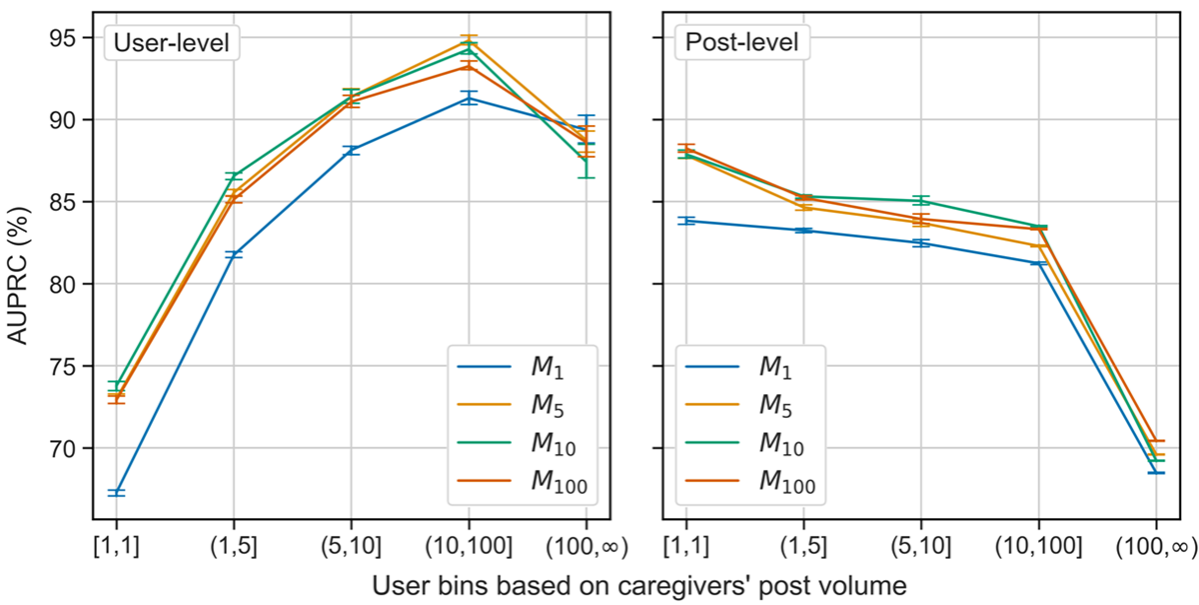
User-level (left) and post-level (right) model performance, in terms of area under the precision-recall curve (AUPRC), of different user bins in the test data set. Note that each user bin defines a post volume range. For example, a user bin of (1, 1) includes caregivers who only published 1 post, whereas a user bin of (100, ∞) includes caregivers who published >100 posts.

Figure 6 illustrates the performance of the AUPRC model for different user bins. As the post volume increased from 1 to 100, all the models had increased AUPRC scores for user-level prediction. However, their AUPRC scores dropped when the post volume of a user exceeded 100. By contrast, there was a consistent decrease in AUPRC scores as the post volume increased for post-level predictions. These opposing observations are primarily owing to the majority voting used for user-level predictions. Although more posts generally improve user-level AUPRC through a majority vote, the dramatic decrease of AUPRC scores in the post-level prediction for the (100,∞) bin negatively affects the user-level prediction. Second, *M_1_* performed worst in most scenarios, suggesting that the sampling of 1 post strategy leads to underfitted models. By contrast, although *M_100_* had the best AUPRC (Table 3 in post-level prediction, it only outperformed other models at a statistically significant level (*t* test; *P*<.001) in bin (100,∞) which contained many more posts than other bins. Notably, M_10_ outperformed M_100_ in a statistically significant manner (*t* test; *P*<.001) in bin (10,100].

#### Error Analysis

To understand the challenge of the prediction tasks, we performed an error analysis of the classification outputs from *M_10_*, which had the best user-level prediction and less bias toward superactive users when compared with *M_100_*.

**Figure 7:**
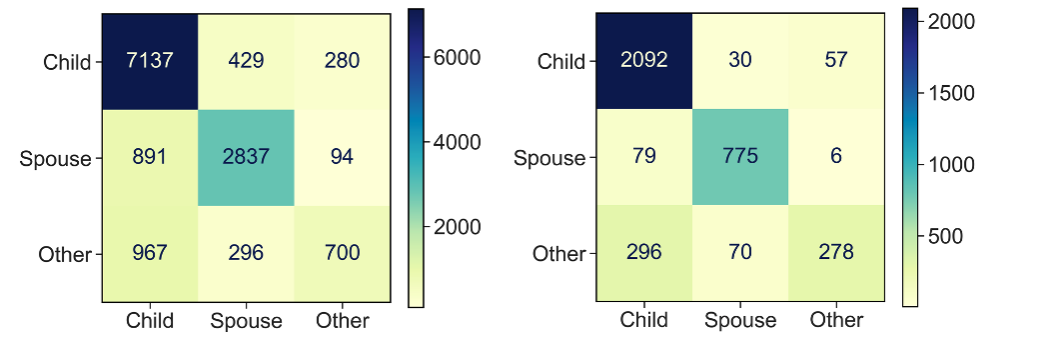
Confusion matrix of the M10 output for post-level (left) and user-level (right) predictions.

Figure 7 depicts the confusion matrix for the entire test for both post-level and user-level predictions. The most difficult task is to distinguish *other* from *adult child*. For example, the misclassified ratio between *adult child* versus *other* and *spouse* versus *other* was (296+57)/(70+6)≈4.6 in user-level prediction and (967+280)/(296+94)≈3.2 in post-level prediction, compared with 59/24≈2.5, the ratio between *adult child* and *spouse* in *D_train_*. After a manual examination, we observed that many caregivers reporting relative relationships with persons living with dementia were children-in-law of persons living with dementia. Here is one such example that is labeled as *other* but predicted as *adult child*: “We moved the MIL to our neighborhood.... She is always mad at FIL.... We would like to know how to ease her mood.” We also observed that most of the 100 randomly selected misclassified posts were short comments expressing gratitude or asking follow-up questions; posts that either mentioned multiple relationships within a long, complex story or did not mention any specific relationship; or posts that asked about balanced living or financial issues. For example, “[NAME], bless you for helping your mom...one person carries a daunting responsibility*.*” was predicted as *adult child* but it was actually composed by an *other* caregiver.

#### Temporal Patterns

**Figure 8:**
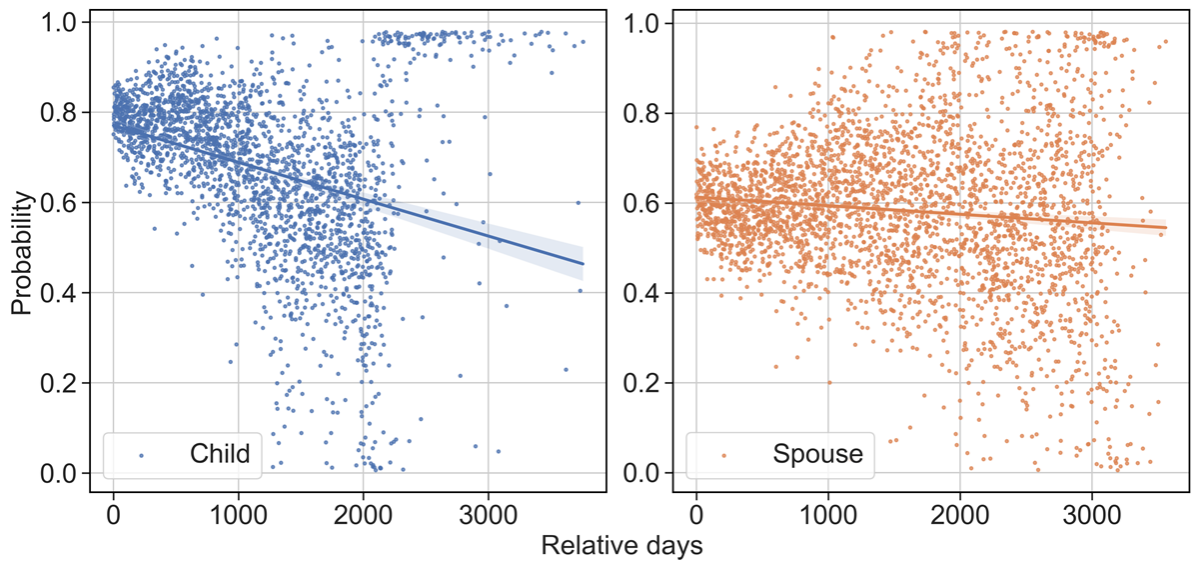
Prediction of caregiver relationship with persons living with dementia over time for adult children (left) and spouses (right).

Figure 8 illustrates the temporal patterns in the prediction probability for the 87,326 posts from 3039 adult children and spousal caregivers in *D_test_*. To improve readability, each point in the figures corresponds to the mean value of the prediction probabilities for a relative day. A linear interpolation and its 95% CI are also shown in each subfigure. There was a decreasing temporal trend for both caregiver types. For *adult child* and *spouse*, the Spearman rank correlation between the probabilities and *relative days* was 0.40 and −0.09 (*P*<.001), respectively. Notably, the longer a caregiver was active in the online community, the more challenging it was to classify the author’s relationship for a post. This suggests that the caregivers shifted their discussion focuses over time. Considering that long-term or veteran users were more likely to comment on other caregivers’ topic threads, some of them might become a specialist or a generalist in this ADRD caregiving online community [57]. However, this phenomenon was less evident among spousal caregivers, indicating their relatively more consistent caregiving experience (eg, caring for persons living with dementia in their daily activities [33]), regardless of the dynamic trajectory of informal caregiving for persons living with dementia [58].

## Discussion

### Principal Findings

This study reports on the online behaviors of adult-child and spousal caregivers in a large online community. There are several principal findings of merit. First, ADRD caregivers are diverse in terms of their relationships with persons living with dementia as well as in the ways they use social support in the online community. For example, the high volume of views in ALZConnected indicates that many online users are “lurkers” or collective knowledge consumers who mostly read online discussions [59]. However, these online caregivers are often neglected by researchers because of a lack of their online voices. Moreover, a majority of the online caregivers who write posts, known as collective knowledge producers, are short-term users. This brevity in platform use is most likely an artifact of their focus on addressing a specific caregiving issue. In recognition of this observation, in addition to the typical goal in the literature to improve caregivers’ long-term well-being [28,60], research should aim to solve the short-term needs of most online caregivers as well.

Notably, there were more adult children than spousal caregivers in ALZConnected. This may be partly because the younger generation is more likely to use the internet. However, spouses tended to publish relatively more posts and actively participate longer in ALZConnected than adult children. Spousal topic threads also received more comments than adult children’s topic threads. Adult-child and spousal caregivers shared many common concerns about issues related to living, sleeping, diet, and finance. Spouses were more likely to discuss other caregiving-related issues such as showering, transportation, disease diagnosis, and the COVID-19 pandemic. This may be due to the close physical bond that a spouse has with the care recipient, the emotional bond, or either a combination of both, as suggested in previous research [7].

Our sentiment analysis showed that both caregiver types experienced an increasing positive sentiment over time in this community, indicating a positive impact on the emotional well-being of caregivers who pursued long-term online support. This is consistent with findings from observational studies that indicate that participating in online communities reduces caregivers’ stress levels [28,30]. However, spousal caregivers had relatively lower sentiment improvement, which, together with their wider range of caregiving discussions (Figures 4 and 5), suggests that they experience more complex challenges than adult-child caregivers.

The best classifier obtained an AUPRC of 81.3% on the user-level prediction. From an algorithmic perspective, our work contributed to an important biomedical research field in estimating online users’ sociodemographic factors from their posts [61,62] by accurately predicting online caregivers’ relationships with persons living with dementia based on their posts. relationships with persons living with dementia based on their posts. Specifically, these factors can be effectively adjusted in any statistical analysis related to online ADRD caregiving discussion. In addition, given that informal dementia caregivers often share their experiences on any online platform they prefer, our classifiers can be applied to help caregivers locate the posts that are published by the same type of caregivers, which will be likely to meet their specific caregiving needs.

The observed decreasing trend in the probability curves on the posting timeline for both caregiver types suggests that online caregivers tended to change or expand their discussion scope over time. This is because these long-term online caregivers are more likely to support other caregivers by composing comments. Community organizers should consider fostering these “veteran users” to sustain the community. Again, the trend of a relatively lower decreasing prediction probability for spousal caregivers provided more evidence that they behave differently from adult-child caregivers. Therefore, tailored strategies should be devised to assist these caregivers based on their relationship type. For instance, spousal caregivers could benefit from an emphasis on daily caregiving matters and emotional support, whereas adult-child caregivers might find greater value in discussions concerning nursing homes, medications, symptoms, and family conflicts. The error analysis showed that the model was more likely to make incorrect predictions between adult children and other caregivers, who, based on a manual examination of the posts, were found to be more likely to be the children-in-law of persons living with dementia. This suggests that children-in-law caregivers, although a small proportion, may share similar caregiving experiences or challenges with adult-child caregivers and should receive attention from the research community and society as well [63].

More broadly, our findings imply that open online communities such as ALZConnected can be leveraged to provide sufficient informational or emotional support to a wide range of informal ADRD caregivers, especially those who live in rural areas where local support services or resources are generally limited [64] and those who hesitate to seek offline support owing to stigma [65]. However, any such translational research is recommended to consider the following issues to ensure fairness and effectiveness of accessing and using online social support: (1) the digital divide caused by either unavailable high-speed internet, low eHealth literacy, or intensive caregiving activities [66]; (2) the misinformation or misconceptions regarding diseases or related treatments [67]; and (3) any potential hatred language that is not uncommon in online environments [68].

### Limitations and Future Work

Despite the merit of our findings, there are several limitations of our study that can serve as the basis for future research.

First, our research focused on a single online community, which may have limited the generalizability of our findings. Therefore, it is necessary to validate our findings using other online communities. However, as ALZConnected is the largest ADRD online community in North America, these findings should be informative to guide future research in this area. Second, we were unable to examine the online behaviors of “lurker” caregivers or include the sociodemographic characteristics (eg, age, gender, sexuality, race, and ethnicity) of online users in our analysis because of the lack of availability of such data in general online environments. Thus, we believe that future research can consider incorporating surveys with users to complement the research findings drawn from merely online posts. Third, we only examined the sentiment encoded in the online discussions. There is clearly an opportunity for investigations that integrate observational research into the study to help measure the psychological well-being of online caregivers, which, together with computational methods, examines how exactly online interactions could improve a caregiver’s well-being. Fourth, we did not evaluate the prediction models in other online communities. This is deemed to be outside the scope of this study, as it requires an intensive survey to collect gold standard relationship information. However, given the promising outcomes of this research, we believe that the built models can be easily adapted to predict the kin relationship between caregivers and care recipients based on the posts in other online ADRD caregiving communities, which can be achieved by using either transfer learning [69] or retraining the models using new annotated data.

### Conclusions

To the best of our knowledge, this is the first study to examine and compare the online behaviors of adult children and spousal ADRD caregivers in a large open online community. Our results show that these 2 caregiver groups exhibited different online behaviors, with spousal caregivers posting more frequently and discussing a wider range of caregiving issues. These observations indicate that spouses experience more complex challenges than adult children. Researchers and community organizers should take into account the heterogeneity of online behaviors, which is likely to be owing to different caregiving experiences, to improve the online experience of different types of caregivers. Further research can be applied to explore how online interactions can address specific caregiving challenges at the topic thread level and benefit both short-term problem-solving and long-term social integration.

## Abbreviations

ADRD: Alzheimer disease or related dementias
AUPRC: area under the precision-recall curve
BERT: Google Pretrained Bidirectional Encoder Representations From Transformers
LIWC: Linguistic Inquiry and Word Count
STM: structural topic modeling
VADER: Valence Aware Dictionary for Sentiment Reasoning

## Data Availability

All the data analyzed in this study are publicly available.
